# Prevalence and Predictors of Self-Reported Swallowing Difficulty in Community-Dwelling Older Adults

**DOI:** 10.3390/jcm15176629

**Published:** 2026-08-27

**Authors:** Lana Fluk, Yuval Nachalon, Amit Shiff, Haya Fogel-Grinvald, Nogah Nativ-Zeltzer

**Affiliations:** 1 Gray Faculty of Medical and Health Sciences, Tel Aviv University, Tel Aviv 6997801, Israel; 2 Department of Otolaryngology, Head and Neck and Maxillofacial Surgery, Tel-Aviv Sourasky Medical Center, Tel Aviv 6423906, Israel; 3 School of Occupational Therapy, Hebrew University, Jerusalem 9190501, Israel

**Keywords:** dysphagia, prevalence, socioeconomic factors, aging

## Abstract

**Objectives:** Dysphagia in older adults is associated with malnutrition, aspiration, and reduced quality of life. This study aimed to estimate the prevalence of self-reported swallowing difficulties among community-dwelling older adults in Israel and to identify demographic, medical, and socioeconomic predictors. **Method:** In this cross-sectional study, 1064 community-dwelling adults aged ≥ 60 years (mean 75.1 ± 7.5 years; 68.1% female) completed an online questionnaire capturing demographics, medical diagnoses, physical activity, income, and place of residence, together with the validated Hebrew and Arabic versions of the Eating Assessment Tool-10 (EAT-10). Dysphagia screening was defined as EAT-10 ≥ 3, and demographic, medical, and socioeconomic predictors were examined using multivariable logistic regression. **Results:** The prevalence of positive dysphagia screening was 29.1%. In the adjusted model, significant independent predictors were older age (odds ratio [OR] = 1.84)), a dysphagia-related medical diagnosis (OR = 3.10), absence of regular physical activity (OR = 2.19), lower income (OR = 1.36), and lower residential socioeconomic cluster (OR = 1.84). Gender, with a mean age of 75.07 years (SD = 7.52), body mass index (BMI), and residential peripherality were not significant after adjustment; the bivariate peripherality effect appeared to be largely mediated by socioeconomic factors. **Conclusions:** Nearly one in three community-dwelling Israeli older adults screened positive for dysphagia. Beyond established medical risk factors, physical inactivity and socioeconomic disadvantage were independently associated with positive screening, suggesting potentially modifiable targets for future study. Findings support integrating swallowing screening into routine geriatric care and developing equity-focused screening initiatives for lower-socioeconomic and peripheral communities.

## 1. Introduction

Dysphagia can lead to significant health consequences, including nutritional deficits, dehydration, aspiration pneumonia, and reduced quality of life. Its prevalence in the general population has been investigated worldwide, yet reported estimates vary considerably, ranging from as low as 5% to over 50% [1,2,3,4,5]. Much of this variation reflects methodological differences across studies, including the populations sampled, the assessment tools and diagnostic thresholds applied, and the size of the study cohorts. These cohorts have ranged widely in size, from 259 participants [1] to more than 51,000 [5].

A key source of this variation is the age composition of the study sample. Because dysphagia becomes more common with age, cohorts weighted toward older adults report substantially higher prevalence than those spanning the full adult age range. In a large U.S. population-based study with a relatively young mean age (46.6 years), about one in seventeen adults experienced dysphagia [4], and Cho et al., (2015) [2], whose sample spanned ages 20–94, similarly reported a low overall rate of 3%. In contrast, studies restricted to older adults have reported far higher estimates—20.1% in adults aged 60 and above [1] and 36.6–47.4% in adults aged 60–91 3.

Socioeconomic factors may also contribute to variability in reported prevalence. In community-dwelling adults, dysphagia has been associated with lower health literacy, particularly a reduced ability to seek medical advice and to access health information and social support [6]. These patterns are consistent with the broader public-health literature linking lower socioeconomic status to a higher burden of chronic disease, poorer access to specialist care, and delayed diagnosis [7]. Dysphagia commonly arises secondary to conditions that show marked socioeconomic gradients, including stroke, neurodegenerative disease, frailty, and head and neck cancer [8]. These disparities are therefore likely to shape both the true prevalence and the recognition of swallowing disorders, as differences in health literacy, symptom awareness, and access to instrumental assessment of swallowing may compound under-identification in disadvantaged groups.

Together, these factors highlight the importance of considering structural and contextual determinants when interpreting variability in dysphagia prevalence across studies. These considerations are particularly relevant in the Israeli context, where marked socioeconomic heterogeneity, cultural and linguistic diversity, and variability in access to healthcare services coexist within a universal healthcare system. This unique combination provides an important opportunity to examine how structural and population-level factors influence dysphagia prevalence and recognition within a single national framework.

Despite growing international prevalence reports, data on dysphagia prevalence in Israel remain limited. Sella-Weiss [9] examined frailty and nutrition in 180 community-dwelling adults aged 65 and older, and reported that 18.3% screened positive on the H-EAT-10. Yet, to our knowledge, no large-scale estimate exists, and the socioeconomic distribution of dysphagia in Israel has not been examined. Understanding the prevalence and characteristics of swallowing difficulties within the Israeli population is essential for healthcare planning, the development of screening and intervention protocols, and the formulation of public health policy. The present study aimed to investigate the prevalence of dysphagia in Israel and identify potential associations with demographic and health-related factors.

## 2. Method

The study was approved by the Tel Aviv University Institutional Review Board (#5193-3).

### 2.1. Participants

This was a cross-sectional survey of community-dwelling adults aged 60 years and older in Israel, conducted between the years 2023–2025. Participants were recruited via email, social media forums, and direct outreach at community centers. Eligibility criteria were age 60 years or older and sufficient proficiency in Hebrew or Arabic to complete the questionnaire. All participants provided informed consent prior to participation.

### 2.2. Procedures

The questionnaire was designed in Qualtrics (Silver Lake, Seattle, WA, USA), an online survey distribution platform. It included items on demographic characteristics, such as body mass index (BMI), medical diagnoses, place of residence, income level, and regular physical exercise, together with the validated Hebrew and Arabic versions of the EAT-10 [10,11,12]. The Eating Assessment Tool (EAT-10) is a validated self-administered questionnaire originally developed by Belafsky et al., (2008) [11] to assess the presence and severity of dysphagia symptoms. A score of 3 or higher is considered suggestive of a swallowing disorder; this threshold was established in the instrument’s original normative validation [11] and has since been widely adopted and confirmed in subsequent validation studies of the EAT-10 as a screening tool for swallowing impairment [13,14]. The Hebrew version of the Eating Assessment Tool-10 (H-EAT-10) has been translated and psychometrically validated for use in Hebrew-speaking adults [10,15] and the Arabic version has been culturally adapted and validated for Arabic-speaking adults [12].

### 2.3. Classification of Residence and Socioeconomic Status

Participants were asked to report their place of residence. Localities were classified according to official indices published by the Israeli Central Bureau of Statistics (CBS) [16]. Socioeconomic status (SES) was determined using the CBS socioeconomic cluster index, which ranks local authorities on a scale from 1 (lowest) to 10 (highest) [17]. In addition, residential peripherality was classified based on the CBS peripherality index, reflecting geographical distance from central population and service centers [16]. According to the CBS, residential areas in Israel are classified using a peripherality index ranging from 1 to 10, where lower values represent more peripheral areas and higher values represent more central areas.

These indices were dichotomized at the scale midpoint (clusters 1–5 vs. 6–10), distinguishing localities below and above the median of the national ranking, an approach used in Israeli health-disparities research [18]. Dichotomization was chosen to aid interpretability and preserve adequate cell sizes, while continuous versions of both indices were retained for the correlation analyses.

Participants reported their monthly individual income in three bands: below 5000 New Israeli Shekel (NIS), 5000–10,000 NIS, and above 10,000 NIS. These thresholds correspond to approximately USD 1350 and 2700 at the average exchange rate over the study period (~3.7 NIS/USD, 2023–2025), or roughly Int$ 1250 and 2500 in purchasing-power-parity terms [19].

### 2.4. Statistical Analysis

Statistical analysis was conducted using SPSS, Version 31.0. (IBM Corp, Armonk, NY, USA). Associations between dysphagia screening status (EAT-10 ≥ 3 vs. <3) and categorical sociodemographic variables (gender, diagnosis, physical activity, income level, and socioeconomic and peripherality clusters) were examined using Pearson chi-square tests of independence. Effect sizes for significant associations were estimated using odds ratios (ORs).

Relationships between EAT-10 total scores and continuous or ordinal demographic and socioeconomic variables (age, income, socioeconomic cluster, and peripherality indices) were assessed using Spearman correlation coefficients. A multivariable binary logistic regression analysis was performed to examine predictors of positive dysphagia screening (EAT-10 ≥ 3), reporting odds ratios and 95% confidence intervals (CIs). Missing data were handled using listwise deletion (complete-case analysis). Bivariate analyses used all available cases for each variable. Frequencies of missing values for specific variables are detailed in Table 1. The significance level was set at α = 0.05.

## 3. Results

### 3.1. Participants

The sample included 1064 participants aged 60 years and older, with a mean age of 75.07 years (standard deviation [SD] = 7.52). Of the participants, 68.1% were female and 31.9% were male. Of the 1064 respondents, 947 (89.0%) completed the Hebrew version of the questionnaire and 117 (11.0%) completed the Arabic version. Monthly individual income was distributed as follows: 28.7% of respondents reported an income below 5000 NIS (~USD 1350), 33.2% between 5000 and 10,000 NIS (~USD 1350–2700), and 27.3% above 10,000 NIS (~USD 2700). Income data were missing for 10.9% of the sample.

### 3.2. Prevalence of Positive Dysphagia Screening

Based on the criterion of an EAT-10 total score ≥ 3, the prevalence of positive dysphagia screening in this sample was 29.1% (310/1064).

### 3.3. Age and Gender

**Figure 1:**
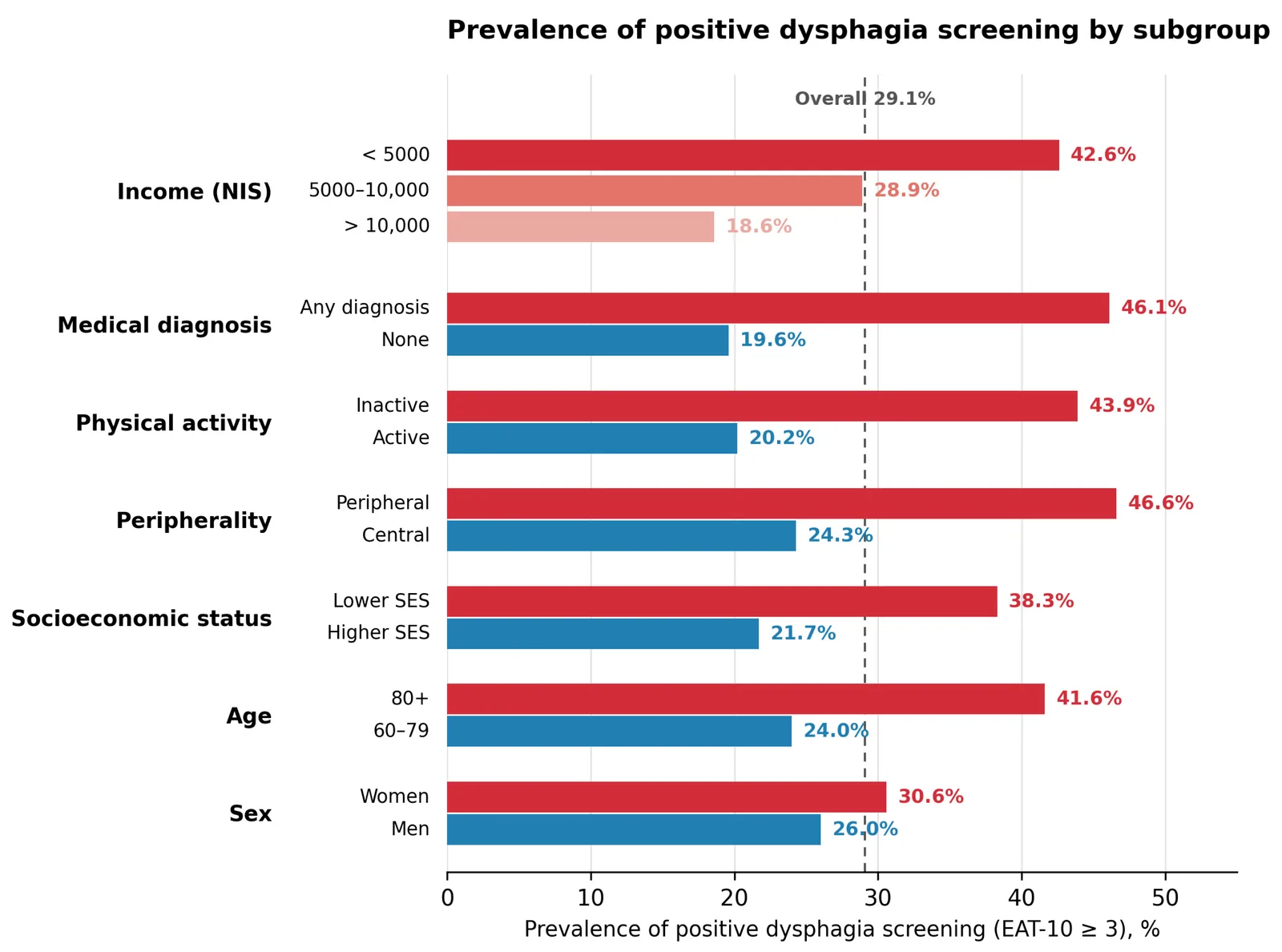
Prevalence of positive dysphagia screening (EAT-10 ≥ 3) within levels of each risk factor among community-dwelling adults aged ≥ 60. The dashed line indicates the overall sample prevalence (29.1%). Income categories are shown as a graded series; all other factors contrast the higher-risk level (red) with the comparison level (blue).

Age was positively correlated with EAT-10 scores (Spearman’s ρ = 0.214, *p* < 0.001), indicating higher symptom severity with increasing age. The association between gender and dysphagia screening status was examined using a Pearson chi-square test of independence. Dysphagia (EAT-10 ≥ 3) was identified in 26.0% of men and 30.6% of women (Figure 1). The analysis indicated that the association between gender and dysphagia screening status was not statistically significant, χ^2^(1) = 2.43, *p* = 0.119.

### 3.4. Medical Conditions

A Pearson chi-square test revealed a significant overall association between specific medical diagnosis category and EAT-10 classification, χ^2^(6) = 81.907, *p* < 0.001. Post hoc comparison indicated that neurological disorders, esophageal disease, recurrent heartburn and stroke were each significantly overrepresented among participants with positive dysphagia screening (EAT-10 ≥ 3) compared to those without dysphagia (*p* < 0.05) (Table 2). In contrast, the proportions of participants with lung diseases and those with a history of surgeries and/or radiation therapy to the head and neck did not differ significantly between the two groups. When medical diagnosis was dichotomized (any diagnosis vs. none), a significant association with positive dysphagia screening was observed, χ^2^(1) = 66.865, *p* < 0.001. Among participants with no relevant medical diagnosis, 19.6% screened positive for dysphagia, compared to 46.1% of those with at least one diagnosis. The odds of screening positive for dysphagia were approximately 3.5 times greater for participants with a medical diagnosis than for those without (OR = 3.496, 95% CI: 2.568–4.760).

### 3.5. Physical Activity

Positive dysphagia screening (EAT-10 ≥ 3) was identified in 20.2% of respondents who engage in regular physical activity and 43.9% of those who do not. The analysis indicated that the association between physical activity and dysphagia screening status was statistically significant, χ^2^(1) = 67.86, *p* < 0.001, OR = 3.088.

### 3.6. Dysphagia and Regional Socioeconomic Status

Correlation analysis revealed a significant negative association between the socioeconomic ranking of place of residence and EAT-10 scores (Spearman’s ρ = −0.18, *p* < 0.001), indicating that residents of lower socioeconomic areas reported more severe swallowing symptoms. A significant association was found between SES cluster (0 = clusters 6–10; 1 = clusters 1–5) and positive dysphagia screening, χ^2^(1) = 35.03, *p* < 0.001, OR = 2.237. Positive screening was more prevalent among participants from lower socioeconomic clusters (38.3%) compared with those from higher socioeconomic clusters (21.7%).

### 3.7. Dysphagia by Region: Central Versus Peripheral Areas

Correlation analysis revealed a significant negative association between the peripherality ranking of place of residence and EAT-10 scores (Spearman’s ρ = −0.21, *p* < 0.001), indicating that residents of more peripheral areas reported more severe swallowing symptoms.

A chi-square test of independence was conducted to examine the association between peripherality cluster (0 = clusters 6–10; 1 = clusters 1–5) and EAT-10 classification. The analysis revealed a statistically significant association between the variables, χ^2^(1) = 43.59, *p* < 0.001, OR = 2.716. Participants from central areas showed a higher proportion of low EAT-10 scores (75.7%) compared to participants from peripheral areas (53.4%). Conversely, a greater proportion of participants from peripheral areas were classified with elevated EAT-10 scores (46.6%) compared to those from central areas (24.3%).

### 3.8. Dysphagia and Income

The association between income level and dysphagia screening status was assessed using a Pearson chi-square test of independence. A significant association was observed between income level and positive dysphagia screening, χ^2^(2) = 41.06, *p* < 0.001. The prevalence of positive screening was highest among participants with lower income levels and decreased progressively with increasing income. Specifically, positive screening was identified in 42.6% of participants earning less than 5000 NIS (~USD 1350) and in 18.6% of those earning more than 10,000 NIS (~USD 2700).

### 3.9. Regression Analysis

A multivariable logistic regression analysis was conducted to examine predictors of the outcome variable. After listwise deletion, the final regression model included 808 participants with complete data on all predictors. Gender, age, BMI, physical activity, medical diagnosis, income level, peripherality cluster, and socioeconomic cluster were all entered into the regression as predictors. The final model was statistically significant, χ^2^(8) = 126.7, *p* < 0.001, Nagelkerke R^2^ = 0.21, and correctly classified 76.4% of cases.

The results indicated that age was a significant predictor (B = 0.612, *p* < 0.001), with the older age group (80+) associated with increased odds of the outcome (OR = 1.84) compared to the younger group (60–79). Medical diagnosis was also a strong significant predictor (B = 1.131, *p* < 0.001; OR = 3.10) as well as not engaging in regular physical activity (B = 0.786, *p* < 0.001; OR = 2.19). In addition, having a lower income level (with the income category variable coded such that higher numerical values indicate lower income; B = 0.310, *p* = 0.010; OR= 1.36) and belonging to a lower socioeconomic cluster (vs. higher; B = 0.608, *p* = 0.001; OR = 1.84) were significantly associated with increased odds of the outcome.

**Figure 2:**
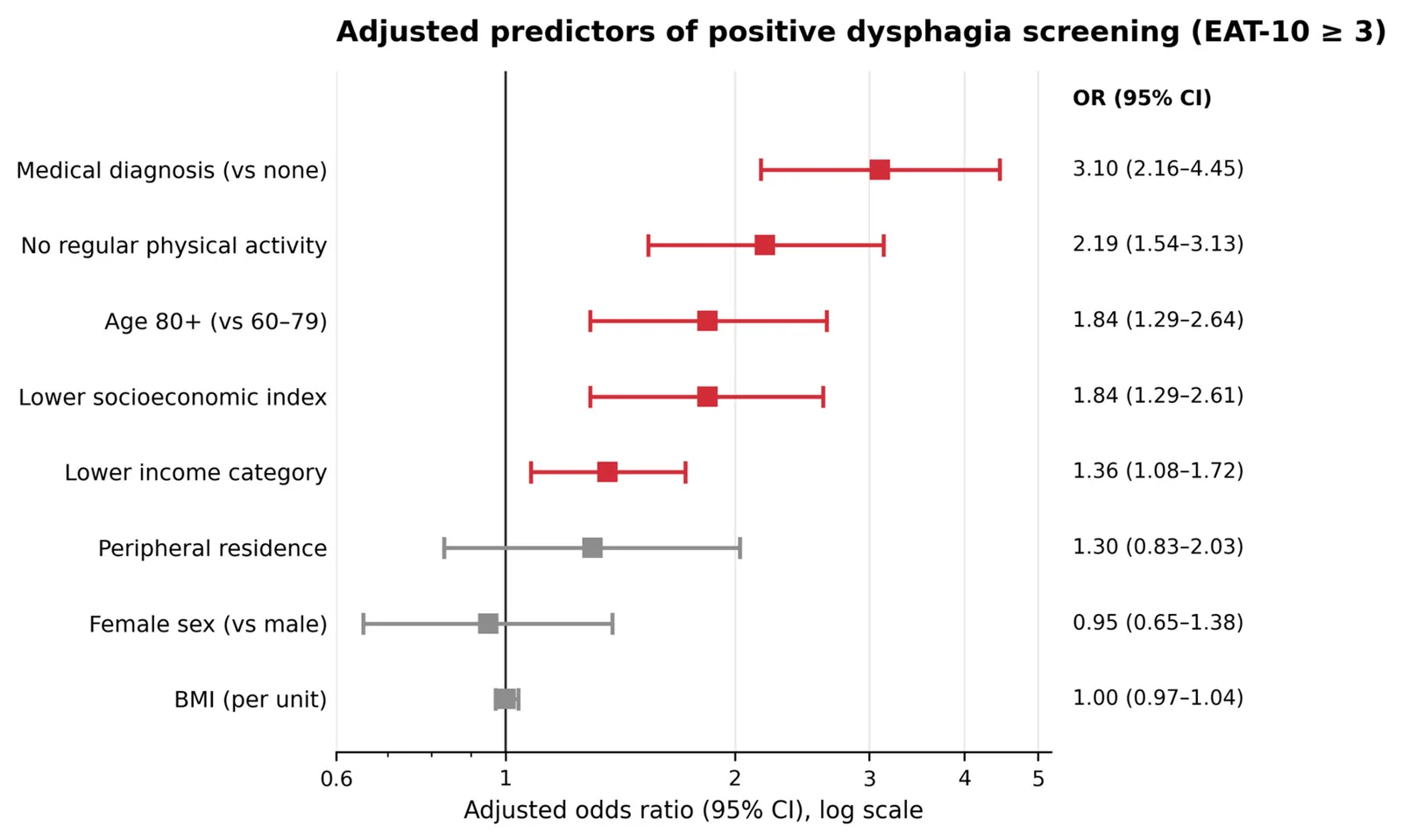
Forest plot of the adjusted ORs from the final regression, on a log scale with the OR = 1 reference line, significant predictors in red and non-significant in gray, plus OR (95% CI).

In contrast, gender (Male vs. Female), BMI, and peripherality cluster (Peripheral vs. Central) did not reach statistical significance (*p* > 0.05). The results of the regression are presented in Table 3 and Figure 2.

## 4. Discussion

To our knowledge, this is the first study to estimate self-reported dysphagia prevalence in a large, bilingual sample of community-dwelling older adults in Israel and to examine its socioeconomic correlates. Using the EAT-10 (cut-off ≥ 3), 29.1% of community-dwelling older adults screened positive for dysphagia, with screening status independently associated with age, medical diagnosis, physical inactivity, and socioeconomic position.

The 29.1% estimate falls within the range reported internationally for community-dwelling older adults, although cross-study comparison is constrained by methodological heterogeneity. It exceeds the ~18% pooled estimate of a recent meta-analysis [20] but falls within the range of studies reported by studies using the EAT-10 specifically, such as 25.1% among independent older adults in Japan [21], 36.6% in India [3], and considerably lower rates in Chinese community surveys [22,23]. Differences in care-dependency, age distribution, and cultural response patterns may contribute to this variability. A previous Israeli study using the H-EAT-10 reported a lower rate (18.3%) [9], which may reflect that study’s exclusion of participants with known dysphagia at enrollment, as well as differences in recruitment. Lower rates more generally typically derive from single-item screening or broader age ranges [2,24]. Our relatively high estimate may reflect both the older age of the cohort (mean 75.1 years) and the sensitivity of the EAT-10 as a multi-item instrument.

Age independently predicted positive screening: adults aged 80 and older had nearly twice the odds of dysphagia as those aged 60–79 (OR = 1.84). Whether risk continues to rise with age within older populations remains debated- some studies report a persistent age gradient [21,25], whereas others did not or find a difference or attribute elevated symptom scores to health status rather than age itself [26,27]. That age remained significant after adjustment for medical diagnosis suggests it captures cumulative, partly subclinical decline related to sarcopenia, polypharmacy, and reduced physiologic reserve.

Gender was not associated with screening status, consistent with some prior reports [2] though not others that found higher rates among women [6,28]. The absence of a gender effect may reflect the age composition of the sample, as sex-related anatomical and hormonal influences on swallowing may vary across the lifespan [29].

A medical diagnosis was the strongest predictor of positive screening (OR = 3.10), consistent with the established links between dysphagia and neurological, cerebrovascular, and esophageal disease [8]. This reinforces the value of systematic swallowing screening in patients with known predisposing conditions.

Physical inactivity was likewise strongly and independently associated with positive dysphagia screening (OR = 2.19), in line with literature linking sarcopenia and physical deconditioning to swallowing impairment [30,31]. Regular activity is associated with preserved skeletal muscle mass, including the muscles of deglutition, together with cardiorespiratory fitness and resistance to frailty, which may partly account for the association observed here [32]. Physical activity may therefore warrant investigation as a modifiable factor in longitudinal and interventional studies.

Socioeconomic position was independently associated with positive screening after adjustment for age, diagnosis, and physical activity: both lower income (OR = 1.36) and residence in a lower socioeconomic cluster (OR = 1.84) remained significant predictors, and bivariate gradients were pronounced (e.g., 42.6% among those earning <5000 NIS vs. 18.6% among those earning >10,000 NIS). These gradients echo findings from the United States, where lower income, public insurance, and unemployment predicted self-reported dysphagia [33], and from South Korea, where lower education and perceived economic hardship increased risk [28]. Several mechanisms may contribute Part of the association may reflect disease burden, as lower socioeconomic status carries a higher prevalence of dysphagia-causing conditions such as stroke, neurodegenerative disease, and head and neck cancer [7]. However, because socioeconomic status remained associated with dysphagia after adjustment for medical diagnosis, additional pathways independent of disease are likely involved. Reduced access to specialist and instrumental assessment can lead to underdiagnosis [8], a particular concern given documented center-periphery disparities in specialist care in Israel [34], while lower health literacy may impair recognition of, and help-seeking for, swallowing difficulty [6]. Together, these point to a need for structural rather than purely individual-level approaches to prevention.

Notably, peripherality of residence was significant in bivariate analysis (OR = 2.72) but not once socioeconomic cluster and income were added to the model, suggesting its association operates largely through socioeconomic pathways rather than geographic distance itself. Because peripheral communities tend to have lower socioeconomic rankings and fewer healthcare resources [16,34], addressing socioeconomic disparity may prove more effective than geographically targeted intervention alone.

Several limitations should be noted. Dysphagia was assessed by self-report (EAT-10) rather than clinical or instrumental evaluation, which may overestimate or underestimate prevalence. Although the EAT-10 is well validated under classical test theory [11],item response theory and Rasch analyses in both clinical samples [35,36,37] and population-based studies [38] have identified psychometric limitations, including item redundancy, disordered rating-scale thresholds, differential item functioning, and floor effects. Our prevalence estimate should therefore be read as a screening-level indicator of self-reported swallowing difficulty rather than a precise measure. Additionally, the cross-sectional design precludes causal inference. Finally, participants were recruited through convenience sampling via email, social media, and community centers. This approach may over-represent individuals who are digitally connected and socially engaged. Self-selection could also influence prevalence estimates if respondents with swallowing concerns were more inclined to participate.

## 5. Conclusions

To our knowledge, this study provides the first large-scale estimate of dysphagia prevalence in Israel and underscores the contribution of socioeconomic and lifestyle factors to swallowing health. Future work should confirm these findings with objective assessment in population-based samples, examine longitudinal risk trajectories, and test whether physical activity promotion and community-based screening, particularly in peripheral and lower-socioeconomic communities, can reduce dysphagia and its socioeconomic gradient.

## Figures and Tables

**Table 1:** Demographic and clinical characteristics.

		*n*	%
Gender	Female	725	68.1
	Male	339	31.9
Age	60–79	754	70.9
	80+	310	29.1
Income (NIS)	Below 5000	305	28.7
	5000–10,000	353	33.2
	Above 10,000	290	27.3
	Missing	116	10.9
Medical diagnosis	None	708	66.5
	Neurological disorder/disease	44	4.1
	Esophageal disease	18	1.7
	Lung diseases	23	2.2
	Surgeries and/or radiation therapy to the head and neck	42	3.9
	Recurrent heartburn requiring pharmacological treatment	83	7.8
	Stroke	44	4.1
	Missing	102	9.6
Engage in regular physical activity	Yes	663	62.3
	No	401	37.7
Peripherality index	Central (6–10)	832	78.2
	Peripheral (1–5)	232	21.8
Socioeconomic index	Higher socioeconomic status (6–10)	589	55.4
	Lower socioeconomic status (1–5)	475	44.6

**Table 2:** Medical diagnoses by EAT-10 category.

Medical Diagnosis	EAT-10 0–2 *n* (%)	EAT-10 ≥ 3 *n* (%)	Total *n* (%)
None	569 (80.6)	139 (54.3)	708 (73.6)
Neurological disorder/disease	19 (2.7)	25 (9.8)	44 (4.6)
Esophageal disease	5 (0.7)	13 (5.1)	18 (1.9)
Lung disease	15 (2.1)	8 (3.1)	23 (2.4)
Surgery and/or radiation therapy to the oral cavity, pharynx, larynx, or neck	26 (3.7)	16 (6.3)	42 (4.4)
Recurrent heartburn requiring pharmacological treatment	51 (7.2)	32 (12.5)	83 (8.6)
Stroke	21 (3.0)	23 (9.0)	44 (4.6)
Total	706 (100.0)	256 (100.0)	962 (100.0)

Values are presented as *n* (% within EAT-10 category). EAT-10 ≥ 3 indicates elevated patient-reported swallowing symptoms.

**Table 3:** Logistic regression model for factors associated with positive dysphagia screening.

Variable	OR	95% CI	*p* Value
Gender (Male vs. Female)	0.95	0.65–1.38	0.771
Age (80+ vs. 60–79)	1.84	1.29–2.64	0.001
BMI	1.00	0.97–1.04	0.984
Regular physical activity (No vs. Yes)	2.19	1.54–3.13	<0.001
Medical diagnosis (Yes vs. No)	3.10	2.16–4.45	<0.001
Income category ^a^	1.36	1.08–1.72	0.010
Peripherality index (Peripheral vs. Central)	1.30	0.83–2.03	0.247
Socioeconomic index (Lower vs. Higher)	1.84	1.29–2.61	0.001

Values are odds ratios (ORs) and 95% confidence intervals (CIs) from a multivariable logistic regression model. The dependent variable was positive dysphagia screening, defined as EAT-10 ≥ 3. ^a^ Variable was coded such that higher values indicate lower income.

## Data Availability

The datasets generated during and/or analyzed during the current study are available from the corresponding author on reasonable request.
